# Striking a balance: analyzing unbalanced event-related potential data

**DOI:** 10.3389/fpsyg.2015.00555

**Published:** 2015-05-01

**Authors:** Roni Tibon, Daniel A. Levy

**Affiliations:** ^1^Baruch Ivcher School of Psychology and Sagol Unit for Applied Neuroscience, The Interdisciplinary CenterHerzliya, Israel; ^2^Cognition and Brain Sciences Unit, Medical Research CouncilCambridge, UK

**Keywords:** mixed-effects models, repeated-measures ANOVA, unbalanced data, event-related potentials, EEG/ERP

## The problem of unbalanced data

The cognitive events examined in many event-related potentials (ERPs) studies do not occur in a neural vacuum, and separating the signals of interest from the brain's background electrical activity generally requires averaging multiple EEG segments of a condition of interest (Luck, 2005). In addition to within-subject averaging, the vast majority of ERP studies are based on across-subject grand average data, i.e., group waveforms representing the means of subjects' averaged waveforms, with statistical significance examined by comparing variance between conditions of interest with variance between participants. Using this approach may not always portray a valid picture. Consider, for example, the following experimental paradigm: in a study of episodic associative memory, participants encoded 120 pairs of stimuli (unrelated object picture pairs in the unimodal task, and unrelated environmental sound-object picture pairs in the crossmodal task). At test, cue pictures were presented to probe recall of the associated picture (in the unimodal task) or sound (in the crossmodal task). ERPs were time-locked to the onset of the cue, and sorted *post-hoc* into recall-success and recall-failure trials (for details of the procedures, see Tibon and Levy, 2014a). This experimental design poses several challenges to the conventional grand-averaging method. First, since the assignment of trials to experimental conditions is based on participants' responses, it is quite likely that the data will be unbalanced (that is, an unequal number of trials in each condition). Therefore, signal-to-noise ratio and variance can vary significantly between experimental conditions. Second, since experimental conditions are mutually dependent (i.e., a participant who had 100 recall-success responses can only have 20 recall-failure responses), participants who were very successful (having a low number of recall-failure trials) or very unsuccessful (having a low number of recall-success trials) in performing the task are likely to be excluded due to an insufficient number of trials for addressing signal-to-noise ratio (SNR) challenges in one of the experimental conditions.

## What can be done differently

To cope with these problems, instead of calculating averages of averages and examining the statistics with repeated-measures ANOVA, we recommend direct examination of ERPs of all trials available in each experimental condition from all subjects, using approaches such as Mixed-effects Models analysis. This method can be considered a generalization of GLM, but uses maximum likelihood estimation instead of sum of squares decomposition. The model is considered “mixed” as it includes two types of statistical effects: (1) fixed effects for which data has been gathered from all levels of the factor(s) of interest, and (2) random effects, assumed to be uncorrelated with the independent variables. Accordingly, the subject is included as a random factor, and inter-individual differences in EEG amplitude dynamics are modeled as a random intercept, which represents an individual “baseline,” in addition to being affected by the fixed factors.

We are not the first to use Mixed-effects Models in analyzing electrophysiological data. More than a decade ago, Bagiella et al. (2000) suggested that this approach has advantages over traditional techniques for EEG data analysis. Baayen et al. (2008) expanded these models to include crossed-random effects for subject and item, and demonstrated that this method deals with common problems of the traditional GLM analysis (unbalanced data, missing values, and non-sphericity). A growing number of EEG studies have employed this method (e.g., Davidson and Indefrey, 2007, 2011; Wierda et al., 2010; Saliasi et al., 2013; Chow et al., 2014), infrequently, alongside the traditional ANOVA (e.g., Janssen et al., 2011). Nonetheless, our design, in which experimental conditions are mutually dependent, provides a unique case-study for systematic comparison between these analyses.

## Mixed-effects models analysis of the example data

In this section, we describe the mixed-effects analysis that was performed on nine electrode clusters, in a time window ranging from 200 to 350 ms post-cue presentation (additional analyses can be found in Tibon and Levy, 2014a). The random factor in our model was subject identity. The fixed part of the model included the task factor (unimodal, crossmodal), the recall-success factor (success, failure), and two spatial location factors: anteriority (anterior, central, posterior) and laterality (left, midline, right). The fixed part of the model further included all possible interactions between the fixed factors. In this mode of analysis, each observation serves as an element to be modeled; degrees of freedom represent the number of observations, and not the number of participants as in grand-average ANOVA. Inevitably, this increases significantly the degrees of freedom, which at a first glance may suggest an overly liberal criterion. However, as we shall show below, the reliability of the statistical findings is not compromised. Model parameters were estimated with the nlme package of the software R (Pinheiro et al., 2007), freely available at http://www.R-project.org). The key finding of this analysis was a significant task X success X anteriority interaction, *F*_(2,63,405_) = 4.76, *p* < 0.01. Decomposition of this interaction revealed that in anterior locations, unsuccessful trials exhibited more negative deflections compared to successful trials in the unimodal task, *t*_(4974)_ = 9.01, *p* < 0.001, but not in the crossmodal task, *t*_(3884)_ = 1.46, *p* = 0.15.

## Repeated measures ANOVA of the example data

To compare our results with those obtained in conventional statistical analysis, we performed repeated measures ANOVA with the same fixed factors as in our mixed-effects analyses. We ran this analysis on several sub-samples: First, we considered all subjects with at least one trial in each condition, i.e., a sample of 36 participants, which we refer to as our *n* = all sample. This analysis is very liberal in terms of ERP SNR, as it includes participants with extremely low numbers of trials. Therefore, we next ran an analysis including only participants with more than 10 trials in each bin (reducing sample size to *n* = 24), and an additional analysis including only participants who had more than 15 trials in each bin (reducing sample size to *n* = 18). Importantly, in this specific experimental design, eliminating participants with low numbers of trials not only increases SNR for each condition, but since the bins are mutually dependent, also improves the balance between the experimental conditions.

For the *n* = all sample, the results did not differ greatly from the mixed-effects results. However, the key task X success X anteriority interaction was marginal, *F*_(1.58,55.4)_ = 3.04, *p* = 0.067, partial η^2^ = 0.08. When we ran the analysis for the *n* = 24 and the *n* = 18 samples, the more subjects we removed, the more the results converged with the mixed-effects results. Specifically, the task X success X anteriority interaction, which was only marginal in our *n* = all sample, became significant when we used the *n* = 24 sample, *F*_(1.49,34.29)_ = 6.49, *p* = 0.008, partial η^2^ = 0.22, and was even more reliable in our *n* = 18 sample, *F*_(1.47,25.02)_ = 7.23, *p* = 0.006, partial η^2^ = 0.3.

To further analyze the recall-success effect that emerged in frontal locations, we used Bonferroni-corrected pairwise comparisons (in this case, with *p* < ~ 0.008). For our *n* = all sample, this revealed a significant effect of success in the unimodal task (*p* < 0.008), but not in the crossmodal task (*p* = 0.028, which does not survive the correction). Notably, while in the mixed-effects analysis, we did not obtain a recall-success effect in the cross-modal task even when the results were not corrected to control type I error, in the standard ANOVA analysis, when no correction was employed the putative recall-success effect *was* significant, i.e., the ANOVA was potentially more vulnerable to Type I error. A significant difference between recall success and failure trials in the unimodal task was also found in our smaller samples (*p*s < 0.008). However, in these cases, the difference in the crossmodal task was not even marginally significant (*p* = 0.13 in the *n* = 24 sample and *p* = 0.34 in the *n* = 18 sample, prior to Bonferroni correction), paralleling the mixed-effects analysis. We further compared the mean amplitudes of these effects, to make sure that lack of effect in the crossmodal task was not simply the result of reduced statistical power, due to the smaller sample size. We found that this was not the case—the difference in amplitudes for the *n* = all sample was 2.33 μV (SEM = 1.02), but was only 1.11 μV (SEM = 0.71) and 0.84 μV (SEM = 0.85) in the *n* = 24 and *n* = 18 samples, respectively. Thus, the differences were indeed reduced in the more balanced sample, in which participants with small numbers of trials in some bins do not make a disproportional contribution to the grand averages. Again, the convergence with the results obtained by the mixed-effects analysis was greater when we used the more balanced sub-samples.

The dissociation between the presence of a recall-success effect in the unimodal task and its absence in the crossmodal task was more pronounced in the *n* = 24 and *n* = 18 samples than in the *n* = all sample due to two factors: first, the significance of the effect in the unimodal task was stronger (e.g., *p* = 0.005 for *n*=all sample vs. *p* < 0.001 for *n* = 18 sample), and the significance of the effect in the crossmodal task was weaker (*p* = 0.028 for *n* = all sample vs. *p* = 0.34 for *n* = 18 sample). Seemingly, the more balanced sample produces different results that are not due to increased type I or II errors, but are simply more accurate. Notably, the more balanced the sample, the more the results resemble mixed-effects analyses—actual differences become more pronounced, while incidental or marginal differences disappear. Importantly, this similarity between the *n* = 18 sample and the mixed-effects analyses emerged even though in terms of participants included, the mixed-effects is more similar to the *n* = all sample.

## Concluding remarks

We have presented an alternative to the common use of grand averaging and repeated-measures ANOVA in analyzing electrophysiological data. Using several data subsets, we have shown that the more balanced the dataset, the more the results of the two methods converged. Importantly, though, by applying the mixed-effects analysis, we did not have to exclude 12–18 (about half!) participants. Since the division of trials into conditions in our paradigm is done *post-hoc*, many participants will not have enough trials in all conditions to be included in a traditional ANOVA. Those participants can, however, be included in the mixed-effects analysis, which balances the data across the whole sample. The fact that the mixed-effects analysis allows us to include virtually all subjects yields better ecological validity—we can include participants whose performance was very good or very bad, and not just those who were more or less average.

Full analysis of data from all trials can be performed not only with mixed-effects models, but also with regression-based methods offering optimization of the ERP waveforms (e.g., Hauk et al., 2006; Groen et al., 2013; Smith and Kutas, 2015a,b; for a brief review, see Rousselet and Pernet, 2011), as well as hierarchical modeling of single-trials and subjects' data (e.g., Kahn et al., 2010; Gaspar et al., 2011; Bieniek et al., 2012). Whether employed for testing hypotheses in pre-defined locations and time windows (as was done in our case) or to test experimental effects at all electrodes and all time points (as implemented in LIMO EEG by Pernet et al., 2011), data analyses can greatly benefit from the use of these approaches. This is particularly relevant when the number of available trials is limited due to practical concerns, as is the case in most paradigms that assess mnemonic processes.

In our particular design, the questions of unbalanced data and of low number of trials are linked. In other cases, these factors might not entirely overlap. However, the case that is presented here is not an “extreme” case, for which the traditional grand-averaging methodology is inadequate. In fact, many experimental designs carry some inherent potential to be based on unbalanced data. Specifically, whenever experimental conditions are populated on the basis of accuracy, or when the task requires some conditions to be more frequent than others (e.g., mismatch negativity) the data is bound to be unbalanced. Therefore, we have employed this method, alongside traditional GLM methods, in additional studies (Tibon et al., 2014a,b; Tibon and Levy, 2014b). We believe that analyses of unbalanced EEG data can greatly benefit from this approach.

### Conflict of interest statement

The authors declare that the research was conducted in the absence of any commercial or financial relationships that could be construed as a potential conflict of interest.
